# A survey of bees (Hymenoptera, Apoidea) in the Bear River Mountains in northern Utah

**DOI:** 10.3897/BDJ.14.e198070

**Published:** 2026-09-15

**Authors:** Lindsie M McCabe, Byron G Love, Jonathan B Koch, Skyler Burrows, Kelsey Graham, Diana Cox-Foster

**Affiliations:** 1 USDA-ARS Pollinating Insects Research Unit, Logan, United States of America USDA-ARS Pollinating Insects Research Unit Logan United States of America; 2 Utah State University, Logan, United States of America Utah State University Logan United States of America

**Keywords:** Apoidea, bee survey, bee inventory, forest service, public lands, Utah

## Abstract

**Background:**

Bees provide critical pollination services to a diversity of flowering plants, facilitating the function of terrestrial ecosystems and global food production. Here, we present a checklist of bee species collected in conjunction with a larger systematic study in the Bear River Mountains of northern Utah, United States, over the course of three consecutive summers (2020-2022). The survey was conducted on public lands managed by the United States Forest Service. We collected 8,500 bee specimens using pan traps, hand nets and trap nests, representing at least 199 bee species belonging to 33 genera across five families. The checklist produced in this survey is critical for land managers and other stakeholders in developing pollinator management and conservation strategies, especially on public lands.

**New information:**

We present the first published checklist of bees in the Bear River Mountains of northern Utah and document five new species that have not been recorded in Utah before: *Ceratina sequoiae*, *Coelioxys alternata*, *Hoplitis pilosifrons*, *Megachile campanulae* and *Stelis holocyanea*. Notably, this survey did not detect the presence of *Osmia cornifrons*, a non-native solitary bee species that is known from lower elevations in the adjacent Cache Valley. However, two non-native species, *Megachile rotundata* and *Anthidium manicatum* were each represented by an occurrence during our three year survey. Early detection of non-native species provides a baseline for potential range expansion.

## Introduction

Bees play a crucial role in sustaining the delicate balance between ecosystem health and global food production. As pollinators, bees are instrumental in mediating the fertilisation of flowering plants, facilitating the reproduction of a vast array of crops and wild plants (Kearns et al. 1998,Klein et al. 2006). This process is vital for the agricultural production of fruits, vegetables, nuts and seeds, contributing significantly to human nutrition (Gallai et al. 2009), while also directly influencing biodiversity in natural ecosystems (Thakur 2012, Nicholls and Altieri 2012). Unfortunately, bees face numerous stressors, including habitat loss (Biesmeijer et al. 2006, Potts et al. 2010, Goulson et al. 2015), pesticide exposure (Goulson et al. 2015, Guzman et al. 2024, Graham et al. 2024) and interannual variation in weather patterns (Soroye et al. 2020, Graham et al. 2021, McCabe et al. 2021, Kazenel et al. 2024), leading to declines in populations of some species (Cameron et al. 2011, Goulson et al. 2015, Janousek et al. 2023). These challenges highlight the urgency of completing comprehensive bee surveys to document species abundance and diversity.

Utah is characterised by many diverse ecoregions including the vast Central Basins and Ranges of the Great Basin to the west, Colorado Plateaus of the Canyonlands to the east and the Wasatch and Uinta Mountains in between (US EPA 2015). Not surprisingly, this diversity in habitat is predicted to host an equally diverse community of wild bees (Orr et al. 2021, Chesshire et al. 2023). A recently published checklist of the bees of Utah reported over 1100 described species with many more undescribed (Wilson et al. 2025).

This study presents a checklist of bees from three life zones — upland, mountain and high mountain — within the Wasatch and Uinta Mountain Ecoregion of northern Utah (Ramsey and West 2009). Specimens were collected over three years as part of a larger project by the authors investigating the effects of commercial apiaries on public lands (Koch et al. 2021 and Cox-Foster et al. *In Review*). The resulting checklist contributes to a regional inventory of wild bee communities in the western United States, a region expected to have great species richness (Orr et al. 2021), but which has been historically undersampled (Chesshire et al. 2023). Regional and local checklists can enhance our understanding of current and future bee distributions.

## Materials and methods

### Study area

The Bear River Mountains are a subset of the Wasatch Mountains (which, in turn, are a subset of the Rocky Mountains) straddling the north-eastern Utah-Idaho border in the United States of America. Made primarily of limestone and dolomite, elevations range from 1,425 – 3,042 m in elevation. The climate is typical of semi-arid, high-desert mountains of the Intermountain West, with the majority of precipitation coming from winter snows, which melt throughout spring and into early summer (Ramsey and West 2009). Mean temperatures range from -5.3°C in January to 18.2°C in July with an annual average of 710 mm precipitation (PRISM Group, Oregon State University 2014). The plant communities in this study include sagebrush steppe, open meadows and mixed conifer and aspen forests. Pollen and nectar resources for bees are provided by a diverse assemblage of primarily perennial herbaceous forbs and woody shrubs.

**Figure 1.**
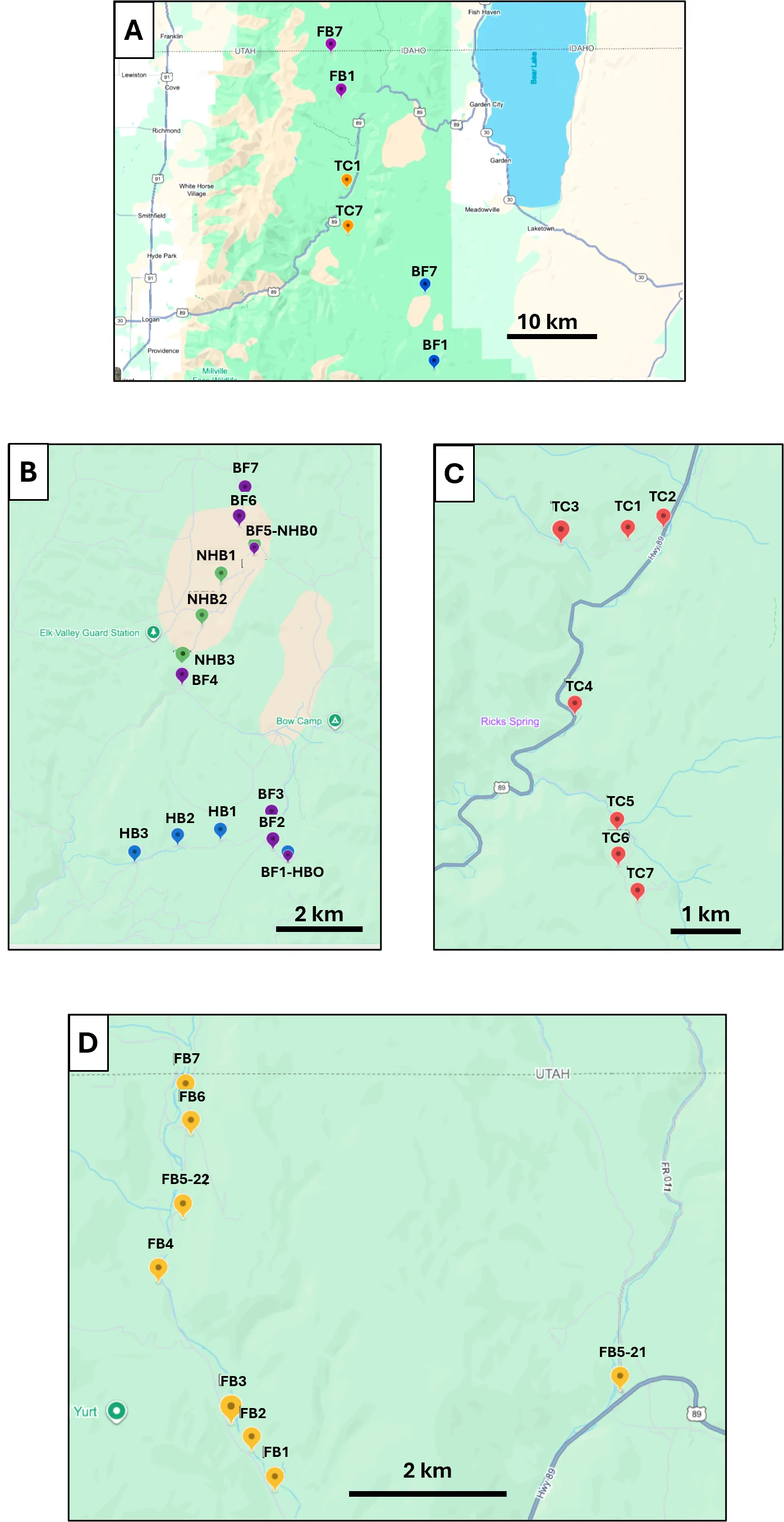
Maps of field sites in 2020 - 2022. **A)** Parent location sites. Blue = Blacksmith Fork, Orange = Twin Creeks, Purple = Franklin Basin; **B)** Blacksmith Fork sites. Blue/green = 2020 sites, Purple= 2021 and 2022 sites; **C)** Twin Creeks sites used in 2021 and 2022; **D)** Franklin Basin sites sites used in 2021 and 2022 (BF5 site in different locations in 2021 vs. 2022). Maps made using Google Earth.

Bees were systematically surveyed in three watersheds managed by the Wasatch-Cache United States Forest Service (USFS); Blacksmith Fork (BS: 2020 - 2166 m), Franklin Basin (FB: 2120 - 2325 m) and Twin Creek (TC: 1793 - 1944 m) (Fig. 1). Watersheds were spaced between 15 and 30 km apart and ranged in elevation from 1793 - 2325 m. In the first year of this study (2020), only bees in Blacksmith Fork were sampled. In years two and three (2021 and 2022), sampling occurred in all three watersheds.

### Collection methods

In each watershed, we established seven 20 m x 20 m plots spaced 0.5-3.5 km apart. The plot sampling schedule was determined by snowmelt (i.e. accessability) and personnel availability. Plots were sampled approximately every two weeks (based on weather and wildfire conditions) starting roughly two weeks after snow melt (10 June, 2020, 20 June 2021 and 21 June 2022) through August (22 August 2020, 19 August 2021, 27 August 2022) for a total of 16 sampling periods (sample dates as follows: 11 June 2020, 23 June 2020, 2 July 2020, 16 July 2020, 30 July 2020, 12 August 2020, 9 - 11 June 2021, 28 - 30 June 2021, 13 - 15 July 2021, 27 - 29 July 2021, 17 - 19 August 2021, 21 - 23 June 2022, 5 - 7 July 2022, 19 - 21 July 2022, 3 - 5 August 2022, 16 - 18 August 2022). In 2021, sampling ended the second week of August due to an extreme drought and began the third week in June in 2022 due to late-melting snow. Bee surveys were conducted in each plot using three methods: hand nets, pan traps and trap nests. Only Blacksmith Fork canyon was sampled in 2020.

Net Collecting: At each of the sites, a 20 m x 20 m plot was established and used throughout the season to conduct sampling. One to three people collected bees off flowers blooming within plots for a total of one-person-hour per sampling date per location (1 person = 1 hour, 2 people = 30 minutes each, 3 people = 20 minutes each). Therefore, each site had net collecting sampling for a total of 16 hours across the three year period. Netting occurred between the hours of 09:00 - 16:00 during conditions favourable to bees (i.e. sunny and warm, no precipitation or excessive wind). Each sampling period was randomised so that plots were sampled at different times time each week.

Pan Collecting: Nine 3.25 oz Solo brand plastic bowls (Solo brand stock number PB6-0099) in three colours (painted with Krylon fluorescent yellow, fluorescent blue and stock white) were filled with soapy water (made up of a 1:1000 solution of Dawn brand blue dish soap in water) and deployed in plots on the same day as net collecting. The bowls were placed in a Y-shape such that an approximately 3 m radius circle was formed as described in McCabe et al. (2019). Pan traps were set out for 4-6 hours between 08:00 and 1630 h. Specimens were sieved and transferred to 4 oz Whirl-Pak bags with 70% ethyl alcohol and kept refrigerated until processing.

Trap Nest Collecting: Nest blocks were used as part of a larger study to determine habitat quality for the managed solitary bee *Osmia bruneri*. We used this opportunity to survey for local cavity-nesting bees utilising the same nest materials. Wood blocks (8 x 8 x 15 cm) with 16 drilled 4 mm holes were fitted with paper straw inserts and placed at each site on metal fenceposts 1 m off the ground facing southeast. Blocks were stocked with eight *O. bruneri* nest straws containing a total of approximately 40 live adults (range 39-43) assuming a 1:1 sex ratio (Frohlich and Tepedino 1986). Nest blocks were deployed for the entirety of each field season and inspected approximately every other week during netting and pan trap sampling. Capped nests were replaced with empty straws to ensure constant nest site availability.

Completed (capped) nests were brought back to the USDA-ARS Pollinating Insect Research Unit (PIRU) in Logan, UT to complete development under local ambient temperature. Nests were x-rayed periodically to confirm the number of cells provisioned and monitor development. Nest cells were removed from the straws at the end of summer and placed into individual gel caps prior to placing in a 4°C cooler for winter diapause. Cells were then incubated at 26°C beginning in mid-spring of the following year and inspected daily for emergence and identification.

### Species identification

Family, genus and subgenus classifications follow Michener (2007), except in some of the *Lasioglossum* subgenera (which follow Gibbs et al. (2012)). Species determinations were made using the following taxonomic keys: Colletidae (Stephen 1954, Snelling 1966a, Snelling 1966b, Snelling 1968, Snelling 1970), Andrenidae (Michener 1935, Timberlake 1956a, Timberlake 1956b, Timberlake 1960, Ribble 1967, LaBerge 1969, Thorp 1969, LaBerge 1973, LaBerge 1980, Laberge and Ribble 1975, Donavan 1977, LaBerge 1977, Bouseman and LaBerge 1978, LaBerge 1986, LaBerge 1985, LaBerge 1989, Shinn 1967) Halictidae (Roberts 1972, Roberts 1973, McGinley 1986, Gibbs 2010), Megachilidae (Mitchell 1933, Michener 1939, Mitchell 1935a, Mitchell 1935b, Mitchell 1936a, Mitchell 1936b, Mitchell 1937a, Mitchell 1937b, Mitchell 1937c, Michener 1938, Sandhouse 1939, Michener 1947, Hurd and Michener 1955, Baker 1975, Grigarick and Stange 1968, Gonzalez and Griswold 2013, Sheffield et al. 2011), Apidae (Hurd and Linsley 1951, LaBerge 1956, LaBerge 1961, Timberlake 1969, Daly 1973, Thorp and Horning 1983, Sipes 2001). In addition, reference specimens from the U.S. National Pollinating Insect Collection (housed at USDA-ARS Pollinating Insect Research Unit (PIRU) in Logan, UT) were used for species verification and identification.

Bees that could be morphologically distinguished from each other, but which lacked taxonomic literature to determine species level, were separated into morphospecies and given alphanumeric titles. When male and female morphospecies could not be associated, male morphospecies were given letters (e.g. *Andrena* sp. A) and female morphospecies were given numbers (e.g. *Lasioglossum* sp. 01). We only included the sex with the larger number of morphospecies in our species counts to avoid falsely inflating the number of species. Specimens with only the genus name and "sp." were left at genus level because the specimen was damaged or compromised in a way that prevented confident species level identification.

Many *Lasioglossum* bees in the subgenus Dialictus were identified only to subgenus due to the difficulty in distinguishing between *Dialictus* species and the lack of comprehensive keys for the western United States. Species in the genus *Sphecodes* and *Nomada* were also only identified to genus level due to the lack of available taxonomic literature for the area.

Specimens were digitised and deposited into the US National Pollinating Insects Collection (NPIC). Records can be downloaded here at the USDA Biocollections database (https://biocollections.ars.usda.gov/collections/) or on GBIF (https://doi.org/10.15468/nqr6jv).

## Checklists

### Checklist of the bees (Apoidea) of the Bear River Mountains

#### Andrenidae

AD0AE8A0-150E-56B0-8519-0FA4607024BE

#### Andrena (Andrena) birtwelli

Cockerell, 1901

302CEA5C-F1EB-55EA-A1F0-3CB69182CC24

#### Andrena (Andrena) saccata

Viereck, 1904

2A8DEA3F-CB61-5D21-85F5-DC5D24505BCA

#### Andrena (Andrena) topazana

Cockerell, 1906

A98F9671-9CDA-5BC9-93C6-4093DFCF400F

#### Andrena (Andrena) vicinoides

Viereck, 1904

BD56D5D3-E915-5183-99DB-5FA5347AFD89

#### Andrena (Cnemidandrena) scutellinitens

Viereck, 1916

3562E268-EFB4-5CF4-8071-0E5B095CFBEF

#### Andrena (Conandrena) cheyennorum

Viereck & Cockerell, 1914

3078B548-CD79-5B80-AF93-5F02B0E360AA

#### Andrena (Diandrena) ablegata

Cockerell, 1922

07A1965A-DC2E-5651-8077-A0C90B94ED62

#### Andrena (Euandrena) astragali

Viereck & Cockerell, 1914

5EDDB564-7E70-52FC-96A3-6C9D8D0EE1D9

#### Andrena (Euandrena) lawrencei

Viereck & Cockerell, 1914

92A6287D-DEEE-5B75-87B2-B28922F32D90

#### Andrena (Euandrena) nigrihirta

Ashmead, 1890

7486EDDD-7D02-504A-A55E-25389C670378

#### Andrena (Euandrena) nigrocaerulea

Cockerell, 1897

AC7ADE78-2486-535D-A63C-896A101E8F5D

#### Andrena (Holandrena) cressonii

Robertson, 1891

CDAB9088-7440-55A9-8F7F-B8419C4C0B9A

#### Andrena (Leucandrena) barbilabris

Kirby, 1802

9766C759-39D1-5FDA-A6D6-A4306174FAB8

#### Andrena (Melandrena) nivalis

Smith, 1853

4F16262F-A212-51D9-9F0B-FCD7ADB6149A

#### Andrena (Melandrena) sola

Viereck, 1916

F74146AF-EC4B-59C1-9188-96D692CE8122

#### Andrena (Melandrena) transnigra

Viereck, 1904

D50F8F41-AE8E-5E18-827F-8376CDD65F3D

#### Andrena (Micrandrena) melanochroa

Cockerell, 1898

62398386-7362-5AD8-A522-C38BC381BA06

#### Andrena (Micrandrena) microchlora

Cockerell, 1922

B5E9CFF5-7B90-5C4F-8091-FDC337DD58D1

#### Andrena (Plastandrena) prunorum

Cockerell, 1896

BAE518C3-FECB-5211-8D94-EEE37B73FA5A

#### Andrena (Ptilandrena) pallidiscopa

Viereck, 1904

2C2C28C0-BE2F-5C01-80D9-BACA88599A40

#### Andrena (Simandrena) angustitarsata

Viereck, 1904

6D8A34FC-6A2C-52C4-87BD-0B6A913B02E2

#### Andrena (Simandrena) pallidifovea

Viereck, 1904

4DCBD8CA-78A4-5EF7-A615-0501FEF0C74A

#### Andrena (Thysandrena) candida

Smith, 1879

DB0BCD60-A1C6-5923-8BF0-C556ED080A0D

#### Andrena (Thysandrena) medionitens

Cockerell, 1902

5751A06A-9591-52A4-A2CF-E9AAA9F50D84

#### Andrena (Thysandrena) w-scripta

Viereck, 1904

0743EA62-D546-5EEE-9A35-FBADE806CC07

#### Andrena (Trachandrena) amphibola

Viereck, 1904

69670A00-A261-5440-BA96-A5F5CE4341F6

#### Andrena (Trachandrena) cleodora

Viereck, 1904

FCAD0290-7C06-5E5C-842F-DA5B2FEC3D8C

#### Andrena (Trachandrena) cyanophila

Cockerell, 1906

D795DBB9-1A51-5272-8CB8-D291D269346F

#### Andrena (Trachandrena) salicifloris

Cockerell, 1897

AC139922-86AB-59BF-ADFF-9A6852473228

#### Andrena sp. 1

A9284551-DEB0-52E3-9EEE-9418D246F027

#### Andrena sp. 2

EAB7698E-8C56-5120-B933-AE361638807D

#### Andrena sp. 3

32D3FBF9-D93B-5D0D-BA50-119E3783B592

#### Andrena sp. 4

45BE6A0C-5249-5CD0-B2CB-90E998B3242C

#### Andrena sp. A

0A6006AE-CACA-5595-9B68-AFC64C1597A8

#### Andrena sp. B

13182B3C-5296-5E99-BCC1-4FD9BE3E955F

#### Calliopsis (Calliopsima) chlorops

Cockerell, 1899

F4B6C2DF-C0B1-591B-95DB-7C365A89EF8D

#### Panurginus atriceps

Cresson, 1878

CAAADF41-E84B-5DF6-A8AB-167B3757F774

#### Panurginus ineptus

Cockerell, 1922

A77E42B7-5925-5C9C-B7BA-BA8787FF9AE0

#### Panurginus torchioi

Cockerell, 1922

9FBBEDC5-4430-5495-A247-EC9B0BEA16FF

#### Perdita (Hexaperdita) ignota

Cockerell, 1896

957827DE-98C6-54C7-BA5E-3F54B2511C05

#### Perdita (Perdita) fallax

Cockerell, 1896

AECE5F27-C875-52C0-BB79-981E95A6AFE1

#### Perdita (Pygoperdita) wyomingensis

Cockerell, 1922

13D4B100-CF18-52C7-9E2A-957062B98EFC

#### Perdita sp. 1

4750F927-A752-54DE-A8BF-7D6DBF022A3A

#### Apidae

8331485C-3174-540C-BC8B-A7E78324AD89

#### Anthophora (Clisodon) terminalis

Cresson, 1869

7F2B0D53-D0AA-5199-B2D9-D4307B3FD1D3

#### Anthophora (Lophanthophora) ursina

Cresson, 1869

3439CBE1-A644-5FD6-BB61-DB02FA7C2CB7

#### Anthophora (Melea) bomboides

Kirby, 1838

E246DD0E-7264-55F1-A580-6BBA74EFFB67

#### Anthophora (Mystacanthophora) urbana

Cresson, 1878

972C9038-E96C-5783-A208-08BC8F3E9656

#### Apis mellifera

Linnaeus, 1758

8686D605-51CB-51BF-8358-81C43E66AF08

#### Bombus (Bombias) nevadensis

Cresson, 1874

2C40BEFA-DBC5-554A-8212-47E3D0E61A60

#### Bombus (Bombus) occidentalis

Greene, 1858

577933EB-0FDB-55ED-877C-24164289BFB3

#### Bombus (Cullumanobombus) rufocinctus

Cresson, 1863

ECC8538E-F72D-59D3-B2FF-81E80D272AF4

#### Bombus (Psithyrus) insularis

Smith, 1861

610C5A83-D215-59C9-A304-4F27DB15275C

#### Bombus (Pyrobombus) bifarius

Cresson, 1878

DAC21EE1-D629-51C3-80C6-3E1A20A34106

#### Bombus (Pyrobombus) centralis

Cresson, 1864

96ACC736-FA5C-51DE-A8C8-7201215CE07F

#### Bombus (Pyrobombus) flavifrons

Cresson, 1863

1A96F15B-1DC4-5B38-B568-A68C93A84B6F

#### Bombus (Pyrobombus) huntii

Greene, 1860

E7863A28-0DCF-534A-A3DA-9DA56D3A5907

#### Bombus (Subterraneobombus) appositus

Cresson, 1878

40E67C9C-560C-535E-A08B-512E6B81D9D7

#### Bombus (Thoracobombus) californicus

Smith, 1854

4B3260DA-C76D-5085-81C5-5B53F2DAB570

#### Bombus (Thoracobombus) fervidus

Fabricius, 1798

E0E71F57-1824-5435-8821-849C77F54828

#### Ceratina (Zadontomerus) acantha

Provancher, 1895

E90966E4-C65D-53D3-88DF-D6DFE770D052

#### Ceratina (Zadontomerus) nanula

Cockerell, 1897

CEEB322E-0387-5DF7-BCB4-262F17AD3FD8

#### Ceratina (Zadontomerus) neomexicana

Cockerell, 1901

A30D1A29-8E3C-57CD-BB8B-086418D4D71E

#### Ceratina (Zadontomerus) pacifica

H.S. Smith, 1907

92A3A109-1521-550B-B643-282CF297C82D

#### Ceratina (Zadontomerus) sequoiae

Michener, 1936

604C46F7-3888-548E-AA23-A0ADBA4B667B

#### Diadasia (Coquillettapis) diminuta

Cresson, 1878

5D3710EC-2A37-557B-9820-E70702BF0A09

#### Diadasia (Coquillettapis) nigrifrons

Cresson, 1878

53F64856-2F0E-54AD-B348-63440CCCC88B

#### Diadasia (Coquillettapis) nitidifrons

Cockerell, 1905

88B91EE1-3718-5E09-BACE-0FEA4627ABFF

#### Diadasia (Coquillettapis) rinconis

Cockerell, 1897

7928041C-4999-593E-A80A-19DB592BCA8E

#### Epeolus spp.

98B106A5-FD58-53C1-B5B6-0273F62D5A6E

#### Eucera (Synhalonia) actuosa

Cresson, 1878

EAC7438C-3139-5C6A-8F9F-C60BC7AF260B

#### Eucera (Synhalonia) edwardsii

Cresson, 1878

64ECA248-C677-581A-A868-AC8F9127EED6

#### Eucera (Synhalonia) frater

Cresson, 1878

060FC87F-84DA-5BEB-9EAA-FB3BF5EFC005

#### Melecta (Melecta) pacifica

Cresson, 1878

35E58E17-271A-560F-8C43-09FF6258C621

#### Melissodes (Callimelissodes) ablusus

Cockerell, 1926

568DA6A1-7BD3-5817-9795-F1CBACC3325E

#### Melissodes (Callimelissodes) lupinus

Cresson, 1878

88B5F386-17C7-5F54-AD46-B2E8A4C52D0E

#### Melissodes (Eumelissodes) microstictus

Cockerell, 1905

D162CE8D-8291-5A85-A64A-37F72420E1A9

#### Melissodes (Eumelissodes) tristis

Cockerell, 1894

CEBA68EB-2BA5-5F1D-A02F-8B76619B16A9

#### Melissodes (Heliomelissodes) rivalis

Cresson, 1872

BA580488-BD98-55DD-A5DD-628034A35FB8

#### Melissodes (Tachymelissodes) dagosus

Cockerell, 1909

02ED2B51-C5EB-53FD-8168-1A44661FC004

#### Nomada spp.

74FDF70D-F285-5D3A-A40D-82003CDA5426

#### Colletidae

D66D5F3C-AD29-5824-9A9C-1CA5C0E7E2A1

#### Colletes consors

Cresson, 1868

E56735F6-66AE-54EC-8D46-C7D59CE7C84B

#### Colletes fulgidus

Swenk, 1904

61547657-E72C-5898-A35A-A659A09B4217

#### Colletes nigrifrons

Titus, 1900

47253138-C960-5070-B4A1-12180FB2AB83

#### Hylaeus (Cephalylaeus) basalis

Smith, 1853

2BCCFED6-AD66-51D0-86C3-8982BC2BDB6E

#### Hylaeus (Hylaeus) annulatus

Linnaeus, 1758

C58E6469-AEEB-5929-BCC0-23781EE4EEA9

#### Hylaeus (Hylaeus) conspicuus

Metz, 1911

ACE3CF33-1CD8-5C7C-9AEA-E03569BA4C90

#### Hylaeus (Hylaeus) rudbeckiae

Cockerell & Casad, 1895

E8FDA4D7-5257-5FA8-8C9E-D0158FE5334A

#### Hylaeus (Hylaeus) verticalis

Cresson, 1869

B7BF406D-E7CA-5978-B54E-F60E2A504402

#### Hylaeus (Paraprosopis) coloradensis

Cockerell, 1896

D85B3DF7-FE7F-5704-9F93-C6C3DF1A180D

#### Hylaeus (Paraprosopis) wootoni

Cockerell, 1896

718B6E5D-94CF-5C86-8A2B-B3E244A19244

#### Hylaeus (Prosopis) episcopalis

Cockerell, 1896

4DE8B0F4-49DA-5A93-BB20-2E0C0EF07F61

#### Hylaeus (Prosopis) modestus citrinifrons

Cockerell, 1896

AB74C547-45BE-5AF9-BE7C-76BA7D3790C5

#### Halictidae

99EA0E76-AEBE-50C4-8FFE-3DABFE9C7511

#### Agapostemon (Agapostemon) angelicus / texanus

5526F720-1C42-5D88-871F-A31CA49270CE

#### Agapostemon (Agapostemon) femoratus

Crawford, 1901

0F675591-3B9C-5AD5-83ED-0EF5A27C99EF

#### Dufourea trochantera

Bohart, 1948

5CE3FC2E-6B3C-5D6A-A997-589438F72776

#### Halictus (Nealictus) farinosus

Smith, 1853

45F2C3E9-1D9C-50B2-B513-29DA7C345BB5

#### Halictus (Odontalictus) ligatus

Say, 1837

9B2DDBD3-83AC-500D-A777-50F4E68F4265

#### Halictus (Protohalictus) rubicundus

Christ, 1791

1C7ACB78-2376-5C29-ADB5-238495F647B8

#### Halictus (Seladonia) confusus

Smith, 1853

D7CF0AC5-35A0-5361-A326-773B89E1D8D6

#### Halictus (Seladonia) tripartitus

Cockerell, 1895

1B3DDD05-B0EB-56AE-80B0-E5C6CC1A6B87

#### Lasioglossum (Dialictus) albipenne

Robertson, 1890

54E0C708-B6FA-5A9E-B8FE-6E4AA783135C

#### Lasioglossum (Dialictus) incompletum

Crawford, 1907

4A50A51E-C8DD-5EE0-A0B0-4B69C0CE4EE1

#### Lasioglossum (Dialictus) sedi

Sandhouse, 1924

78A3E635-071A-5C6E-B5E7-C416B2A69D2F

#### Lasioglossum (Dialictus) spp.

CAD6AC9A-FCB5-5C9E-B4E8-5703DCB8159B

#### Lasioglossum (Evylaeus) cooleyi

Crawford, 1906

CCAAD89B-27BB-56C4-AEC1-7B34CF5F28D3

#### Lasioglossum (Hemihalictus) spp.

4880515A-0861-56C2-86D9-0363ED130CE4

#### Lasioglossum (Lasioglossum) anhypops

McGinley, 1986

076B7032-33A2-5AA7-97F2-6CA9FBBC3DDC

#### Lasioglossum (Lasioglossum) athabascense

Sandhouse, 1933

6ADCF2F0-FB18-58AC-BD07-E99D7B113B1B

#### Lasioglossum (Lasioglossum) sisymbrii

Cockerell, 1895

9F5FE0A8-7E3A-5CA6-84B0-B8783EC44342

#### Lasioglossum (Lasioglossum) titusi

Crawford, 1902

3342D48E-110B-50D1-997F-C9D88B9F8928

#### Lasioglossum (Lasioglossum) trizonatum

Cresson, 1874

CBF11EB5-B775-59FA-82A5-2500A7AA921D

#### Sphecodes spp.

75365EA8-84E7-57A7-9F2B-5194B6BD92AC

#### Megachilidae

B698B1C6-E473-5680-B9D4-5AD42591082C

#### Anthidiellum (Loyalanthidium) robertsoni

Cockerell, 1904

E279894B-DBEE-5083-9017-41179DE8E182

#### Anthidium (Anthidium) atrifrons

Cresson, 1868

B95E54CD-6BD7-525F-8678-6E5BC4C9CC9F

#### Anthidium (Anthidium) manicatum

Linnaeus, 1758

80127D39-E66B-53B2-9614-C16F577E0B95

#### Anthidium (Anthidium) mormonum

Cresson, 1878

49C33133-2CA0-5910-8AFB-21F5B76B8E7D

#### Anthidium (Anthidium) utahense

Swenk, 1914

D41E6A73-4443-5E60-8C7F-51F02BD01E39

#### Anthidium (Callanthidium) formosum

Cresson, 1878

9B099C99-990E-5C4D-A633-B51C3A03095D

#### Ashmeadiella (Ashmeadiella) bucconis

Say, 1837

61D79891-39D4-55EB-8F9C-094CB4F89C33

#### Ashmeadiella (Ashmeadiella) cactorum

Cockerell, 1897

AAEF38A2-FCD0-5AE7-AB1E-35F16FB9DA46

#### Ashmeadiella (Ashmeadiella) californica

Ashmead, 1897

77E74E39-5C2C-5F2F-B3AF-28DCCB60B338

#### Ashmeadiella (Ashmeadiella) cubiceps

Cresson, 1879

A5AAA3B9-5873-57DB-91B4-7F3C7760D12B

#### Ashmeadiella (Ashmeadiella) difugita

Michener, 1939

E672C05E-C85A-5C1A-8D92-FCA0DD7E0D5D

#### Atoposmia (Atoposmia) abjecta

Cresson, 1878

4F0EC0CC-CE5C-56F9-9129-82AB81E04437

#### Atoposmia (Hexosmia) copelandica

Cockerell, 1908

2A9F9C9A-1C7B-5624-8E32-C69BE63DB00B

#### Coelioxys (Boreocoelioxys) rufitarsis

Smith, 1854

B516A835-8FB6-5A9F-A3EA-D6F6412AD204

#### Coelioxys (Synocoelioxys) alternata

Say, 1837

2D509F95-E04F-5D2F-8D8D-4B252FBF3714

#### Dianthidium (Dianthidium) pudicum

Cresson, 1879

113E6AD6-CBBC-56E7-8377-E5E36E1DD654

#### Dianthidium (Dianthidium) singulare

Cresson, 1879

91656E06-6694-5FBF-9B6C-C925CF9E774C

#### Dianthidium (Dianthidium) subparvum

Swenk, 1914

9CF0F949-21AE-55A6-B798-455E23933FF8

#### Dianthidium (Dianthidium) ulkei

Cresson, 1878

CC790C95-0A49-5B93-858A-57FD8A0BBE04

#### Dioxys pomonae

Cockerell, 1910

38F5D655-75B9-53DE-BA31-4824504C3E94

#### Heriades (Neotrypetes) carinatus

Cresson, 1864

D4DC0FC1-F402-517C-888E-19B37E37BA2E

#### Heriades (Neotrypetes) variolosus

Cresson, 1872

69ED31F1-03B8-500D-BE88-8FF105ACD0D7

#### Hoplitis (Alcidamea) albifrons

Kirby, 1837

1ACEF96A-B127-5D63-9476-BFBE1BFBD8EF

#### Hoplitis (Alcidamea) fulgida

Cresson, 1864

6D2092CD-520B-569F-AB49-E8790550FC1B

#### Hoplitis (Alcidamea) grinnelli

Cockerell, 1910

63BDC35C-9CEE-5831-BFC5-C9A7329ED46D

#### Hoplitis (Alcidamea) pilosifrons

Cresson, 1864

057A2E46-F794-5AE7-AE50-FCBD3964C0EC

#### Hoplitis (Alcidamea) producta

Cresson, 1864

92FD979E-3BBE-52D1-A922-502E53D208E3

#### Hoplitis (Alcidamea) sambuci

Titus, 1904

12CF4B5F-D7B2-59A3-ADCB-8F08CBDC77A4

#### Hoplitis (Formicapis) robusta

Nylander, 1848

7E15EE8E-061C-56C9-B0CB-047CD75558C2

#### Hoplitis (Monumetha) louisae

Cockerell, 1934

AEEA0AFD-CA9C-5F1E-99B5-52B0590EF303

#### Megachile (Argyropile) parallela

Smith, 1853

AE5F71B1-D812-525C-9329-FF70C549087A

#### Megachile (Chelostomoides) campanulae

Robertson, 1903

95124E10-6AAA-555B-B492-1A1843D0282B

#### Megachile (Eutricharaea) rotundata

Fabricius, 1787

57408F6E-2A6A-5C70-B2A2-5EAF49A9552A

#### Megachile (Litomegachile) gentilis

Cresson, 1872

35D97E2B-CCFC-5F98-8F05-671020F5278C

#### Megachile (Litomegachile) onobrychidis

Cockerell, 1905

0F6522E1-7307-557C-BEF1-6F2F8ADA8EB5

#### Megachile (Megachile) inermis

Provancher, 1888

969656E1-2060-5932-83DF-65E99D6DC4C5

#### Megachile (Megachile) montivaga

Cresson, 1878

257B1455-852D-5049-9430-9C943D907038

#### Megachile (Megachile) relativa

Cresson, 1878

B2278D1E-7D87-5916-A175-B6048E0645E3

#### Megachile (Megachiloides) manifesta

Cresson, 1879

FAFAAABC-0DA9-5AF7-B9AD-889BF1BFBF33

#### Megachile (Megachiloides) wheeleri

Mitchell, 1927

8130316F-F2FE-505B-95A3-C8669B441E90

#### Megachile (Sayapis) fidelis

Cresson, 1878

5E97214B-30CD-5672-A825-B24BF210B82A

#### Megachile (Sayapis) pugnata

Say, 1837

37DC21FE-3609-5BEC-A8E5-5B18F758DD6F

#### Megachile (Xanthosarus) frigida

Smith, 1853

696C576F-64AF-5003-BA1C-B81947284D5F

#### Megachile (Xanthosarus) gemula

Cresson, 1878

3CFFA61D-028D-52A8-90A4-A7F3FCB88271

#### Megachile (Xanthosarus) melanophaea

Smith, 1853

5545BCF8-8A6E-55E4-B5FF-C6CA0BCE276B

#### Megachile (Xanthosarus) perihirta

Cockerell, 1898

22982D34-0160-530C-AABF-E093B34C6F94

#### Osmia (Cephalosmia) californica

Cresson, 1864

2F6C63F7-1174-5088-96AE-2307DA613D55

#### Osmia (Cephalosmia) grinnelli

Cockerell, 1910

DA0D990E-A972-5AE3-A7B4-C37C2BA814CB

#### Osmia (Cephalosmia) montana

Cresson, 1864

983D239B-F62F-5173-B497-A536636F7DA1

#### Osmia (Cephalosmia) subaustralis

Cockerell, 1900

8D6661CD-08B4-5BD5-A6D0-972982EC0E18

#### Osmia (Chenosmia) trevoris

Cockerell, 1897

B238989A-EE5E-5A35-8C64-D6E1EDD250B0

#### Osmia (Hapsidosmia) iridis

Cockerell and Titus, 1902

354A290F-5DAF-556A-B7A9-3B4C4EC01AB0

#### Osmia (Helicosmia) coloradensis

Cresson, 1878

D0E52B4F-3A64-5909-A12A-5853B3F13C1A

#### Osmia (Helicosmia) texana

Cresson, 1872

0A6DD1A0-BFBB-50E4-877B-E778E50F5F10

#### Osmia (Melanosmia) albolateralis

Cockerell, 1906

0034CBBB-8044-5482-B0B8-05717E678200

#### Osmia (Melanosmia) atrocyanea

Cockerell, 1897

38D6FD64-F85D-5788-BDA4-B84221BB6954

#### Osmia (Melanosmia) bella

Cresson, 1878

992F6735-C1EB-5101-9B14-8749AA979DEA

#### Osmia (Melanosmia) brevis

Cresson, 1864

11209512-B4DF-5F44-AF80-5A179F16EFD7

#### Osmia (Melanosmia) bruneri

Cockerell, 1897

9544D8B5-7516-5F2C-A198-99E30C5E504C

#### Osmia (Melanosmia) bucephala

Cresson, 1864

A7EA5B01-E87C-505B-A462-6F692C56962F

#### Osmia (Melanosmia) calla

Cockerell, 1897

9BFD48C7-F258-5504-A878-12A1F1541DF0

#### Osmia (Melanosmia) ednae

Cockerell, 1907

34575B88-ED0D-5B87-BBDE-A329911B11F2

#### Osmia (Melanosmia) kincaidii

Cockerell, 1897

72961911-2902-54C6-87D6-1F0A96CC3280

#### Osmia (Melanosmia) laeta

Sandhouse, 1924

FF852A7A-E6EA-5920-A113-5A66D31E0BC7

#### Osmia (Melanosmia) longula

Cresson, 1864

C0F4779A-AECA-53AD-97BD-389A955EA7EC

#### Osmia (Melanosmia) melanopleura

Cockerell, 1916

C59C3AC9-F676-5D51-B063-ED138C3429C3

#### Osmia (Melanosmia) sp. aff. indeprensa

Sandhouse, 1939

143F0DF5-7AD9-5F73-A11C-D74250D183AB

##### Notes

new species *O. parkeri*, manuscript in prep

#### Osmia (Melanosmia) nifoata

Cockerell, 1909

013A5D1E-B1E6-5BCD-B14F-E753AB7C70A1

#### Osmia (Melanosmia) nigrifrons

Cresson, 1878

4C0524E1-2890-5D95-ADF5-194EE6AD6319

#### Osmia (Melanosmia) paradisica

Sandhouse, 1924

9F5413A6-682C-5DF7-8D2E-527EA5278BCD

#### Osmia (Melanosmia) phaceliae

Cockerell, 1907

43C1A348-BE2C-5F4C-AC97-C18AFCD114BB

#### Osmia (Melanosmia) pikei

Cockerell, 1907

863B3697-B5DB-509D-838B-896E64C829EB

#### Osmia (Melanosmia) proxima

Cresson, 1864

ADFB8FD6-9CBE-5C91-A681-772342606BC7

#### Osmia (Melanosmia) pusilla

Cresson, 1864

18DB4842-953A-5E64-BE40-51BFCC2950B7

#### Osmia (Melanosmia) raritatis

Michener, 1957

20C35EBC-C42B-5048-9926-63A079D84110

#### Osmia (Melanosmia) sculleni

Sandhouse, 1939

1EF2461D-4353-5FCB-B1D5-DB48C57B624B

#### Osmia (Melanosmia) simillima

Smith, 1853

FF7302BD-13EC-576D-94B6-DACB1673F183

#### Osmia (Melanosmia) tersula

Cockerell, 1912

1014C607-B9C8-57CB-8FC5-EFB4663AF8EE

#### Osmia (Melanosmia) tristella

Cockerell, 1897

9C4991DC-B8B1-5C89-B4FB-FCA5963678CE

#### Osmia (Mystacosmia) nemoris

Sandhouse, 1924

F48E35F3-A14D-58F1-8B0D-567EACCDC9DB

#### Osmia (Osmia) lignaria

Say, 1837

AD9079E7-F66A-56BC-A44F-82CCB6C45B51

#### Stelis (Stelis) ashmeadiellae

Timberlake, 1941

014105B6-7709-53F6-979F-A15640E24DCB

#### Stelis (Stelis) calliphorina

Cockerell, 1911

38C5537C-0490-5A82-95E1-A939C1CA18D8

#### Stelis (Stelis) holocyanea

Cockerell, 1925

4FCB282B-E34F-572A-AC13-D2C46457AF2E

#### Stelis (Stelis) montana

Cresson, 1864

95E142FF-15B3-5F1D-A2CA-DC09B12FF0F8

#### Stelis (Stelis) subemarginata

Cresson, 1878

ADBE0E0F-1039-558F-A65E-94C6400C26BC

#### Stelis (Stelis) sp. 2

6DDAFEBB-744A-5C39-BD2C-B33E1904D702

##### Notes

new species, Manuscript in prep. (Griswold et al.).

## Analysis

**Figure 2.**
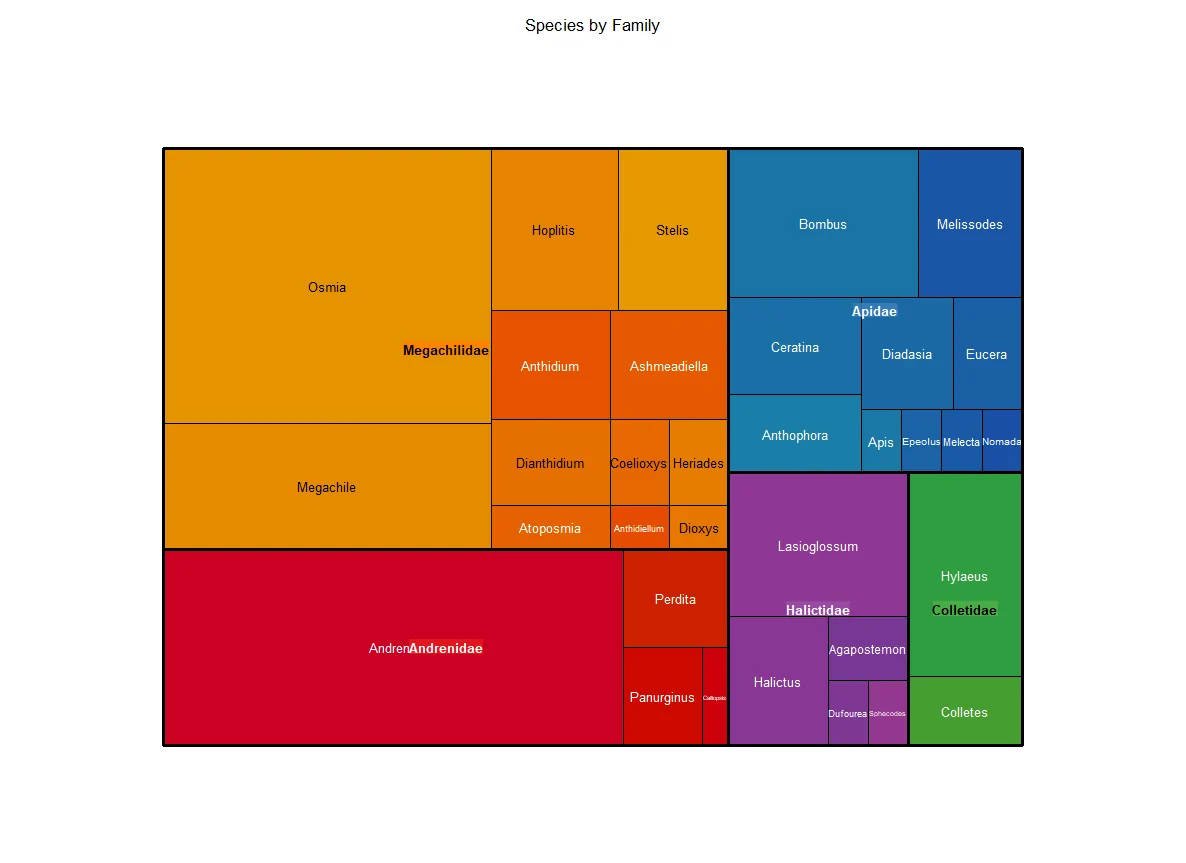
Bee species richness by genus. Box size indicates the proportion of total species represented by each genus. Genera are grouped by family.

Our three-year survey collected 8,500 bee specimens of 199 species or morphospecies belonging to 33 genera and five families (refer to Checklist and *Suppl. material 1*). At the family level, Megachilidae contributed 44% of the species identified in this study (n = 87) representing 25% of bee abundance (n = 2130) (*Fig. 2*). Andrenidae contributed the second highest number of species (21.5%, n = 43), but representing only 5% of bee abundance (n = 430). On the other hand, Halictidae only contributed 10% of the species in this checklist (n = 20), but included 45.5% of the bee abundance (n = 3876) (this ranking would undoubtedly change if all *Lasioglossum* subgenus *Dialictus* were identifiable to species). Apidae ranked third in both species richness (18.5%, n = 37) and bee abundance (20.5%, n = 1743), with a majority of this abundance being *Apis mellifera* (41%, n = 719). Species richness at the genus level was highest in *Osmia* (n = 35) followed by *Andrena* (n = 29), *Megachile* (n = 16), *Bombus* (n = 11) and *Hylaeus* (n = 9) (we did not include *Lasioglossum* in this ranking due to the high number of unidentified specimens). Five of these species are new records to UT not found in *Wilson et al. (2025)* nor documented on GBIF to occur in UT; *Ceratina sequoiae, Coelioxys alternata, Hoplitis pilosifrons, Megachile campanulae* and *Stelis holocyanea*.

**Figure 3.**
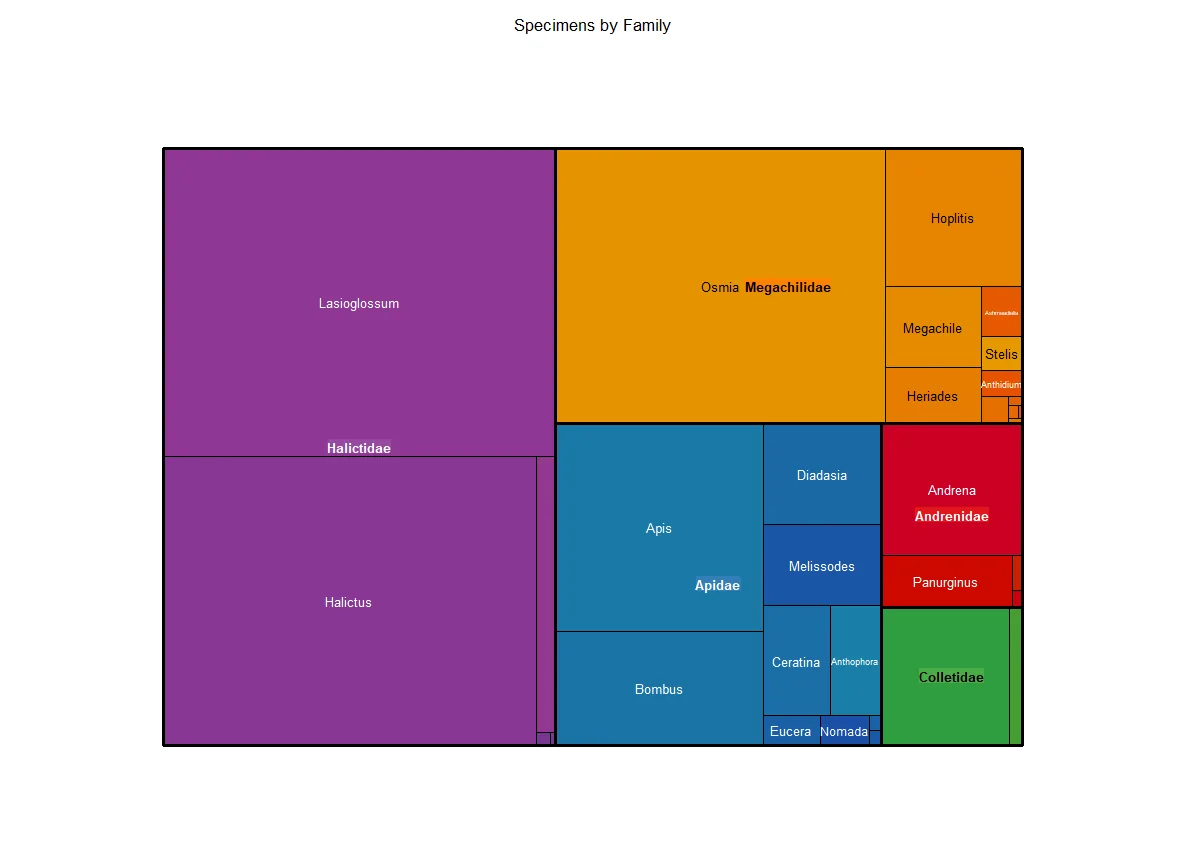
Bee abundance by genus. Box size indicates the proportion of total bees represented by each genus. Genera are grouped by family.

Sweat bees from the family Halictidae, contributed over 45% of the bees collected in this study, primarily from *Halictus tripartitus* (n = 1,556) and the mostly unidentified species in the *Lasioglossum* subgenus *Dialictus* (n = 1189) (Fig. 3). The managed honeybee, *Apis mellifera* (Apidae) made up 8% of the total number of bees collected (n = 719), which is not surprising since six out of our 21 sites had managed apiaries in close proximity to sampling area, followed by *O. bruneri*, the species we stocked in trap nests (n = 685; 8%). There was a total of 48 singleton species (only one individual collected per species) and 15 doubletons. Only two individuals of the common sweat bee, *Agapostomen texanus/angelicus* were collected during this study. This result was surprising as this species complex is documented to be quite abundant in the Intermountain West.

## Discussion

### Comparison of bee distribution

This survey provides one of the most comprehensive assessments to date of bee diversity in a high‑elevation region of northern Utah and fills natural history knowledge gaps of montane bee communities in the Intermountain West. The nearly 200 species documented across three watersheds highlight the richness of bees in the Bear River Mountains and underscores the importance of conducting systematic inventories in regions historically undersampled relative to their predicted biodiversity (Orr et al. 2021, Chesshire et al. 2023). Similar high‑elevation surveys in the Rocky Mountains and adjacent upland regions have also reported high species diversity and strong seasonal turnover, consistent with the patterns observed here (Cane et al. 2013). The dominance of *Osmia*, *Andrena* and *Megachile* aligns with other montane checklists and supports the concept that mountain ecosystems serve as important reservoirs of both early‑season specialists and cavity‑nesting taxa (McCabe et al. 2020, Orr et al. 2021).

Of the five documented species, it is likely that two of these species, *Ceratina sequoiae* and *Hoplitis pilosifrons* are range expansions from what has previously been recorded. *Ceratina sequoiae* is commonly found throughout California, Oregon and Washington, but rarely found east of the Sierra Nevada mountain range. Our collection is the furthest east this species has been found. *Hoplitis pilosifrons*, is commonly found in the eastern and mid-western United States as well as dispersed pockets throughout the west. This is the fist record of it being found in Utah or anywhere in the Great Basin Region. First records of *Coelioxys alternata, Megachile campanulae* and *Stelis holocyanea*, are likely not range expansions, but new records due to challenges in identifying these species. *Coelioxys* and *Stelis* are challenging genera to identify to species and often times only identifed to morphospecies, which leads to data deficiencies at the species level. *Megachile campanulae* is more common in the east, but does occur in the west; however, similarly looking species *M. angelarum* are much more common in the west.

**Figure 4.**
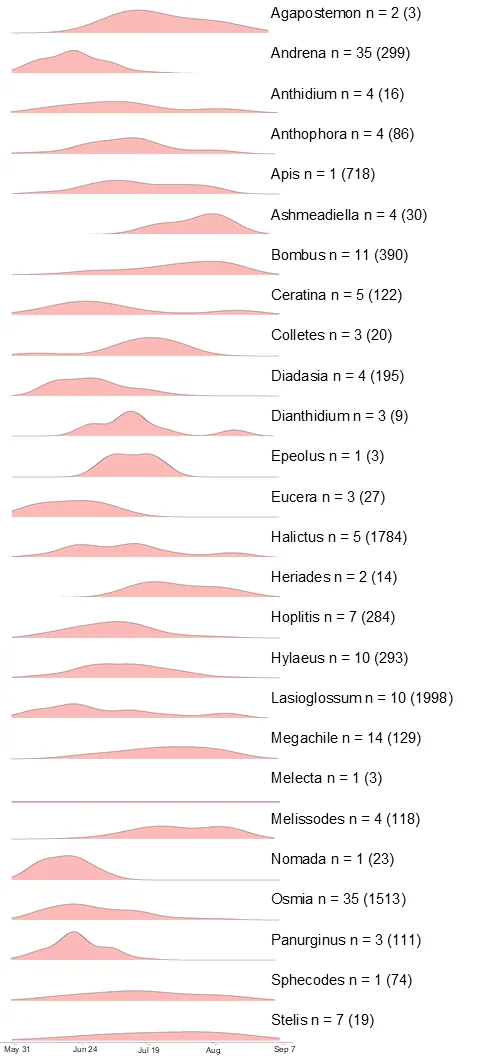
Phenology graphs of each genus, based on data collected from the three year survey. n = species richness and abundance for each genus.

The 33 genera exhibited expected spatiotemporal variation in distribution (Cane et al. 2013). For instance, Fig. 4 illustrates phenological variation amongst bees such that a spring guild of bees includes *Andrena, Diadasia, Eucera, Nomada*, *Osmia, Nomada* and *Panurginus*, a summer guild of bees includes *Ashmeadiella*, *Melissodes* and *Megachile*, while other bees, such as *Bombus, Agapostemon* and *Halictus* were distributed throughout the growing season. Species were also distributed differentially in space (Suppl. material 1); while 37.5% of species were collected in all three watersheds (n = 75); additionally, there were 32 unique species in Blacksmith Fork, 26 in Twin Creeks and 20 unique species in Franklin Basin.

### Limitations of collection methods

Collection methods differed in their contribution to species richness. Unsurprisingly, pan traps contributed the most specimens and species (n = 5457 and 164 species), followed by net sampling (n = 2222 and 148 species) and trap nests (n = 821 and 14 species), this pattern being consistent with other literature on collection method biases (Montero‐Castaño et al. 2022). Collection methods also differed in the contribution of unique species (species only collected from one sampling method), with pan traps again making the highest contribution (n = 47), followed by net sampling (n = 42) and only three unique species, *Hylaeus verticalis*, *Osmia iridis* and *O. pikei*, from trap nests (Suppl. material 1).

The low contribution of trap nests to the bee diversity reported in this study is likely explained by their primary purpose in the larger research objectives, habitat assessment for a single bee species, *O. bruneri*, rather than sampling for cavity-nesting species diversity. Not surprisingly, *O. bruneri* was the most abundant species reared from trap nests (n = 685), likely reflecting the reproductive success of stocked nests. The PIRU has been managing a population of *O. bruneri* for over 20 years, occasionally augmenting the population with nests collected from nearby watersheds. Consequently, we cannot differentiate between stocked and wild *O. bruneri*, although it is probable that our results include both. The low species diversity can also be explained by the limited type of nesting material used; our trap nests consisted of only a single hole size (4 mm) using a paper straw insert, designed specifically for *O. bruneri* (*Frolich and Parker 1983*; B. Love & L. McCabe, unpublished), which limits the proportion of the local cavity-nesting community that could be sampled. For instance, cavity-nesting *O. lignaria* and *O. californica* were collected using nets and pan traps, but not trap nests, probably due to their need for larger diameter holes (Tepedino and Torchio 1989). Individuals of another large cavity-nesting bee species, *Megachile pugnata*, were often observed trying to enter the trap nests with mixed success and sometimes dying with their head lodged in the nest entrance (Suppl. material 2). Adult body size dictates what size nesting cavities bees will use (i.e. larger bees nest in larger holes and smaller bees nest in smaller holes) (Strickler et al. 1996). However, to some degree, there is variation in what constitutes suitable nesting sites. For instance, *Osmia lignaria* are known to nest in holes from 5–8 mm; however, 6 mm is their preferred cavity size (Pinilla‐Gallego et al. 2022). Here we documented that *M. pugnata*, which usually nest in 8 – 10 mm cavities (Frolich and Parker 1983), successfully nested in 4 mm straws and produced six offspring. This is the first documented case of this species successfully nesting in this small of a cavity, indicating that nesting resource availability may play a role in where these bees choose to nest.

### Non-native species

Establishment of non-native species has been an ecological concern for decades. Some introductions of non-native bee species, such as *Osmia taurus* and *Anthidium manicatum*, have been shown to outcompete and/or displace native species and lead to their decline (Gibbs and Sheffield 2009, Graham et al. 2018, LeCroy et al. 2020, Gutierrez et al. 2023). Conversely, the introduction of the Eurasian leafcutting bee, *Megachile rotundata*, in the 1940s for widespread use as a managed commercial pollinator of alfalfa seed crops across North America has had no documented impact on native species (Pitts-Singer and Cane 2011). Interestingly, in the adjacent Cache Valley, there are records of multiple non-native species, according to the USGS exotic species list: *Osmia cornifrons*, *Megachile rotundata* and *Anthidium manicatum*. However, over three years of sampling, *A. manicatum* and *M. rotundata* were each collected only once. This shows that these species appear to be more closely tied to developed areas at this time. Outside of our survey, only one record of *M. rotundata* was recorded in the early 2000s and one record of *A. manicatum* in 1962 in our study area. This suggests that these bees may be present, but are not readily abundant in the non-urbanised montane landscape. However, since our survey ended in 2022, there have been five additional iNaturalist records documenting *A. manicatum* from 2023-2025 in this study area (iNaturalist contributors 2026). This underscores the importance of surveys in detecting changes in non-native species' abundances and distributions.

### Conclusions

This survey provides a foundational understanding of bee diversity in the Bear River Mountains and highlights the ecological value of sustained, systematic monitoring across elevational and habitat gradients. By documenting nearly 200 species, including six newly recorded in Utah and establishing baseline information on the presence and rarity of non-native bees, this work strengthens regional pollinator inventories and offers critical insight for land managers tasked with maintaining healthy ecosystems on public lands. The pronounced differences in species richness across families, watersheds and collection methods underscores the importance of using diverse sampling approaches when assessing bee communities. Additionally, by integrating multiple sampling approaches within a coordinated design, we were able to simultaneously generate a comprehensive regional checklist and fulfil the objectives of a complementary experimental study. Together, these findings not only contribute to closing long‑standing data gaps for an undersampled region, but also creating a benchmark for tracking future changes in bee distributions, community composition and potential expansions of non-native species in northern Utah’s montane landscapes.

## Supplementary Material

75953D26-59EF-5597-BFD5-36432E99913F 10.3897/BDJ.14.e198070.suppl1 Supplementary material 1 Bee occurrence and collection method Data type Table Brief description Bee species collected during a three-year survey (2020-2022) in the Bear River Mountains of northern Utah, United States of America. Numbers of species column reflects bee species richness by Family and Genus. All other columns are species abundance values by Family, Genus, Collection method (hand netting, pan trap or trap nest) and Location (BF = Blacksmith Fork, FB = Franklin Basin, TC = Twin Creek). File: oo_1731237.xlsx https://binary.pensoft.net/file/1731237 McCabe, L.; Love, B.

1A7AF46D-E2E7-578A-AB5F-477ED06B1089 10.3897/BDJ.14.e198070.suppl2 Supplementary material 2 Deceased *Megachile pugnata* Data type Image Brief description Deceased *Megachile pugnata* with head lodged in 4 mm cavity nest. File: oo_1351350.jpg https://binary.pensoft.net/file/1351350 McCabe
